# Deconstructing white matter connectivity of human amygdala nuclei with thalamus and cortex subdivisions in vivo

**DOI:** 10.1002/hbm.23639

**Published:** 2017-05-17

**Authors:** Aslan Abivardi, Dominik R. Bach

**Affiliations:** ^1^ Department of Psychiatry Psychotherapy and Psychosomatics, Psychiatric Hospital, University of Zurich Zurich 8032 Switzerland; ^2^ Division of Clinical Psychiatry Research Psychiatric Hospital, University of Zurich Zurich 8032 Switzerland; ^3^ Neuroscience Center Zurich, University of Zurich Zurich 8057 Switzerland; ^4^ Wellcome Trust Centre for Neuroimaging and Max Planck UCL Centre for Computational Psychiatry and Ageing Research, University College London London WC1N 3BG United Kingdom

**Keywords:** connectome, DWI, neuroanatomy, structural connectivity, paraventricular, probabilistic tractography

## Abstract

Structural alterations in long‐range amygdala connections are proposed to crucially underlie several neuropsychiatric disorders. While progress has been made in elucidating the function of these connections, our understanding of their structure in humans remains sparse and non‐systematic. Harnessing diffusion‐weighted imaging and probabilistic tractography in humans, we investigate connections between two main amygdala nucleus groups, thalamic nuclei, and cortex. We first parcellated amygdala into deep (basolateral) and superficial (centrocortical) nucleus groups, and thalamus into six subregions, using previously established protocols based on connectivity. Cortex was parcellated based on T1‐weighted images. We found substantial amygdala connections to thalamus, with different patterns for the two amygdala nuclei. Crucially, we describe direct subcortical connections between amygdala and paraventricular thalamus. Different from rodents but similar to non‐human primates, these are more pronounced for basolateral than centrocortical amygdala. Substantial white‐matter connectivity between amygdala and visual pulvinar is also more pronounced for basolateral amygdala. Furthermore, we establish detailed connectivity profiles for basolateral and centrocortical amygdala to cortical regions. These exhibit cascadic connections with sensory cortices as suggested previously based on tracer methods in non‐human animals. We propose that the quantitative connectivity profiles provided here may guide future work on normal and pathological function of human amygdala. *Hum Brain Mapp 38:3927–3940, 2017*. © **2017 Wiley Periodicals, Inc.**

## INTRODUCTION

Across species, the amygdala functions as a neural hub, interconnecting and influencing distant regions of the brain [Todd and Anderson, 2009]. Reflecting its central location in functional brain circuits, alterations in amygdalo‐cortical networks are associated with several neuropsychiatric conditions such as autism [Kleinhans et al., 2008], schizophrenia [Ford et al., 2015; Vai et al., 2015] and anxiety disorders [Baur et al., 2013; Greening and Mitchell, 2015]. More specifically, the amygdala is integral to the modulation of attention, perception and memory, as well as higher‐order cognitive functions [Phelps and LeDoux, 2005], and is prominently known for its role in storing threat memories [Bach et al., 2011b; LeDoux, 2000; Maren, 2001]. The primate amygdala in particular has furthermore been linked to social cognition, reward learning, extracting information from faces [Adolphs, 2010], and prioritizing threat information [Bach et al., 2015]. Based on functional considerations, a direct projection from thalamic pulvinar nucleus to amygdala [LeDoux, 1998; Tamietto and De Gelder, 2010] has been proposed to rapidly relay visual threat cues. More recently, communication between the central amygdala and the paraventricular thalamus (PVT) was demonstrated in rodents and shown to be essential in the formation of threat memory and control of fear responses [Do‐Monte et al., 2015; Penzo et al., 2015]. To date, an analogous pathway in humans has not been established. This functional importance of the amygdala motivates the detailed study of its anatomical connections.

In non‐human animals, the amygdala is extensively connected to cortex [Freese and Amaral, 2009; McDonald, 1998]. A particular architecture has been suggested by McDonald [1998] in which direct afferents from primary sensory cortices exist only for olfactory, gustatory and visceral modalities, while auditory, visual and somatosensory information is conveyed through distinct, modality‐specific, cortical cascades. Additionally there is firm evidence for direct connections from the auditory thalamus to the amygdala [Romanski and LeDoux, 1992], while direct connections with visual thalamus, that is, pulvinar, have been suggested [LeDoux, 2000]. The connectivity profile is proposed to differ between amygdala subnuclei [Carmichael and Price, 1995; McDonald, 1998; Pitkänen, 2000]. While earlier models were mainly based on qualitative or semi‐quantitative tracing methods, more recently quantitative mapping of non‐human primate amygdala connections with temporal and prefrontal cortices [Ghashghaei and Barbas, 2002; Ghashghaei et al., 2007] and the thalamic reticular nucleus [Zikopoulos and Barbas, 2012] have confirmed intricate differences in subnuclear connectivity. Contrasting a wealth of information on non‐human animals and in particular rodent species, information concerning the structural connectivity of the human amygdala is still sparse and non‐systematic, notably also concerning projections from and to thalamus.

Probabilistic tractography based on diffusion‐weighted magnetic resonance imaging (DWI) provides a valuable tool to estimate the trajectories of neural connections at the macroscale in vivo [Jbabdi et al., 2015]. Here, we capitalize on this method to characterize the connectivity of amygdala nuclei with individual thalamic and cortical areas, including important thalamic subregions such as the pulvinar and PVT. We manually segmented the amygdala from surrounding tissue, and automatically parcellated it into deep and superficial nucleus groups using a previously developed protocol [Bach et al., 2011a], which is based on clustering of voxel‐by‐voxel connectivity with a combined orbitofrontal cortex (OFC) and temporal pole (TP) target region. While this parcellation scheme reduces interpretability of connections from individual amygdala nuclei to the two cortical regions used in the parcellation, connectivity to other cortical and thalamic regions remains unharmed.

Parcellation of the thalamus into subregions was performed with an established winner‐takes‐all method based on voxel‐to‐region connectivity [Behrens et al., 2003a; Johansen‐Berg et al., 2005; Traynor et al., 2010]. Cortex was automatically parcellated based on T1‐weighted MR images [Desikan et al., 2006; Destrieux et al., 2010; Fischl et al., 2004].

## MATERIALS AND METHODS

### Participants, Data Acquisition and Preprocessing

Preprocessed MRI datasets of 50 healthy volunteers (28 female, age 22–35 years, mean 30 years) were obtained from the human connectome database (http://humanconnectome.org) [Van Essen et al., 2013]. We selected the first 50 subjects from the WU‐Minn HCP Data‐80 unrelated subjects release, which excluded persons with diagnosis of neurological or psychiatric disease, or structural abnormalities in their MRI images. Data were collected on a customized Siemens 3T Connectome Skyra Scanner at Washington University. DWI scanning parameters included a multiband factor of 3 [Moeller et al., 2010] an isotropic voxel resolution of 1.25 mm, FOV = 210 × 180 mm, 270 diffusion directions distributed over 3 shells with *b*‐values of 1,000, 2,000 and 3,000 s/mm^2^, diffusion times of Δ = 43.1 ms and *δ* = 10.6 ms, *G*_max_ = 97.4 mT/m, TE = 89 ms, TR = 5.5 s, FA = 78°, BW = 1,488 Hz/pixel, ES = 0.78 ms and a scan time of 55 min [Sotiropoulos et al., 2013; Van Essen et al., 2013]. Scans were repeated in two phase‐encoding directions to enable correction of susceptibility artefacts. Structural T1‐weighted images were acquired using a 3D MPRAGE sequence [Mugler and Brookeman, 1990] at an isotropic voxel resolution of 0.7 mm, FOV = 224 × 224 mm, TR = 2,400 ms, TE = 2.14 ms, TI = 1,000 ms, FA = 8°, BW = 210 Hz/pixel, ES = 7.6 ms and with optimized contrast parameters to ensure accurate cortical distinction [Glasser et al., 2013]. Data preprocessing was performed using HCP structural and diffusion preprocessing pipelines [Glasser et al., 2013; Sotiropoulos et al., 2013]. These consisted of a model‐based protocol, which concurrently corrects for head motion as well as susceptibility and eddy‐current artefacts [Andersson and Sotiropoulos, 2015, 2016]. More specifically, susceptibility artefact correction is enabled through acquisition of pairs of diffusion images with reversed phase encoding (PE) direction. These PE‐reversed images are combined to estimate an off‐resonance field. Eddy‐current distortions were then assessed using information from diffusion scans acquired with opposing diffusion gradients and integrated with the susceptibility induced off‐resonance field and a model of subject motion using an iterative Gaussian Predictor.

Subcortical and cortical structures were identified from T1‐weighted images using a modification [Glasser et al., 2013] of the “recon‐all” pipeline in FreeSurfer Version 5.2 (http://surfer.nmr.mgh.harvard.edu/) [Dale et al., 1999; Fischl et al., 2001, 1999a, 1999b, 2002, 2008; Segonne et al., 2005]. Automated volumetric subcortical parcellation assigned anatomical labels to individual voxels based on a probabilistic atlas estimated from a manually delineated training set [Fischl et al., 2002]. High consistency between automated volume estimation of thalamus using FreeSurfer and manual estimation has been independently reported [Keller et al., 2012]. Automated cortical parcellation assigned anatomical labels to the cortical surface based on a combination of geometric information and data derived from a manually segmented training set [Desikan et al., 2006; Destrieux et al., 2010; Fischl et al., 2004]. This provided 74 masks of cortical gyri and sulci per hemisphere. A full list of these segmentations can be found in Destrieux et al. [2010].

### Probabilistic Tractography

The FMRIB Software Library (FSL) [Jenkinson et al., 2012] was used for probabilistic tractography. All tractography was done in native diffusion space, and was transformed to T1 image space for post‐processing and display in SPM8 and Matlab. All masks used for tractography were warped from structural to diffusion space using the transformation matrix included in the HCP dataset. In a first step we used the FSL function bedpostx [Behrens et al., 2007] which relies on Markov‐chain–Monte‐Carlo (MCMC) sampling to estimate probability distributions over fibre orientations at each voxel of each subject. Specifically we used a multishell model based on continuous gamma distribution of diffusivities [Jbabdi et al., 2012] and a maximum of three fibre directions at each voxel to account for crossing tracts. This algorithm also corrects for gradient non‐linearities [Sotiropoulos et al., 2013]. To reduce computation time, we used a GPU adaptation of the software, which accelerates estimation through parallel processing [Hernandez et al., 2013]. Probabilistic tractography was performed with the FSL function probtrackx [Behrens et al., 2003b, 2007] with default settings (number of samples = 5,000, curvature threshold = 0.2, step length = 0.5 mm, number of steps = 2,000) on the estimated distribution derived from the multishell data. This function simulates tracts by starting in a seed voxel, drawing a sample from the previously calculated distributions of diffusion directions, proceeding to the adjacent voxel indicated by the sample, and continuing repetitively until ending in a pre‐defined termination area. The number of traces ending in a given termination voxel is recorded. Tractography was run separately (using specific termination masks) between deep or superficial amygdala segmentations as seed, and 6 ipsilateral thalamus segmentations as target; between amygdala segmentations as seed and ipsilateral paraventricular thalamus (PVT) as target; and between amygdala segmentations as seed and 74 ipsilateral cortical segmentations as target. Termination masks to stop tracts were defined as combined cortex, thalamus, brainstem and cerebellum as well as a midline plane. This served to suppress indirect connections and limited tractography to the ipsilateral hemisphere. The termination mask for tractography to the PVT did not include the thalamus because of inconsistencies in mask overlap across subjects.

### Amygdala Parcellation

**Figure 1:**
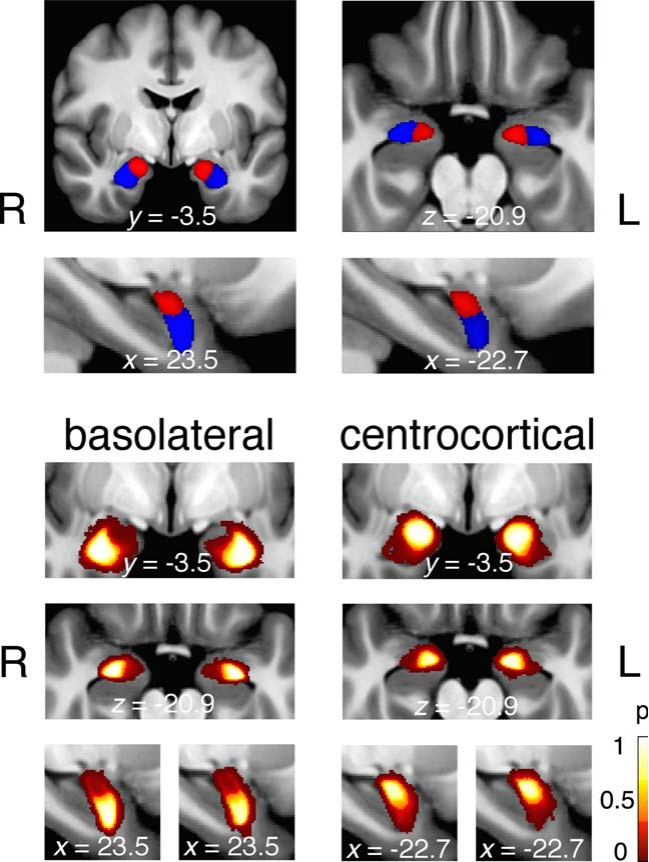
Coronal, axial and sagittal views of group‐level amygdala parcellations in MNI space. Maximum probability maps in upper panels are thresholded at P = 0.3 (superficial cluster red, deep cluster blue). Unthresholded probability maps for basolateral and centrocortical clusters in lower panels.

Manual delineation of the amygdala was performed on individual T1‐weighted images using FSLView in comparison against schematic tables of an anatomical atlas [Mai et al., 2016], following the protocol from Bach et al. [2011a]. Using the temporal horn of the lateral ventricle, hippocampus, optical tract and sulcus semiannularis as guiding landmarks, we proceeded from posterior to anterior in coronal slices, marking external boundaries of the amygdala. We then corrected the resulting mask first in sagittal, then axial and once more in coronal orientation. Mask boundaries were smoothened using SPM8 functions *spm_erode* and *spm_dilate*. Mean volume ± SD of the seed masks was 1,043 ± 182 mm^3^ for left, and 992 ± 169 mm^3^ for right amygdala, which is similar to our previous report [Bach et al., 2011a]. Amygdala parcellation into deep (including basolateral) and superficial (including centromedial and cortical) nucleus groups was implemented using our previously established protocol [Bach et al., 2011a]. This approach is based on k‐means clustering of voxel‐to‐voxel connectivity from entire amygdala to combined temporal pole and orbitofrontal cortex. The target area was created by merging respective regions defined in FreeSurfer. Tractography was performed between amygdala as seed and ipsilateral combined TP‐OFC mask as target using probtrackx with default settings. A termination mask stopped tracts entering thalamus, brainstem or bilateral target, and thus suppressed alternate pathways. The cross‐correlation matrix of all seed voxels' connectivity pattern was entered into automated k‐means clustering [Behrens and Johansen‐Berg, 2005; Johansen‐Berg et al., 2004] using the algorithm by Hartigan [1975] and 200 iterations with a fixed number of two clusters. Cluster contiguity was enhanced using seed voxel location as a constraint for the clustering. About 99.94% ± 0.18% of voxels were grouped into two spatially contiguous clusters across the group. Remaining non‐contiguous voxels were iteratively reassigned to the surrounding cluster. Mean volume ± SD of the clusters was 488 ± 126 mm^3^ for left, and 500 ± 118 mm^3^ for right deep cluster; 555 ± 141 mm^3^ for left, and 491 ± 112 mm^3^ right superficial cluster. Group probability maps for resulting amygdala segmentations are shown in Figure 1.

### Thalamus Parcellation

**Figure 2:**
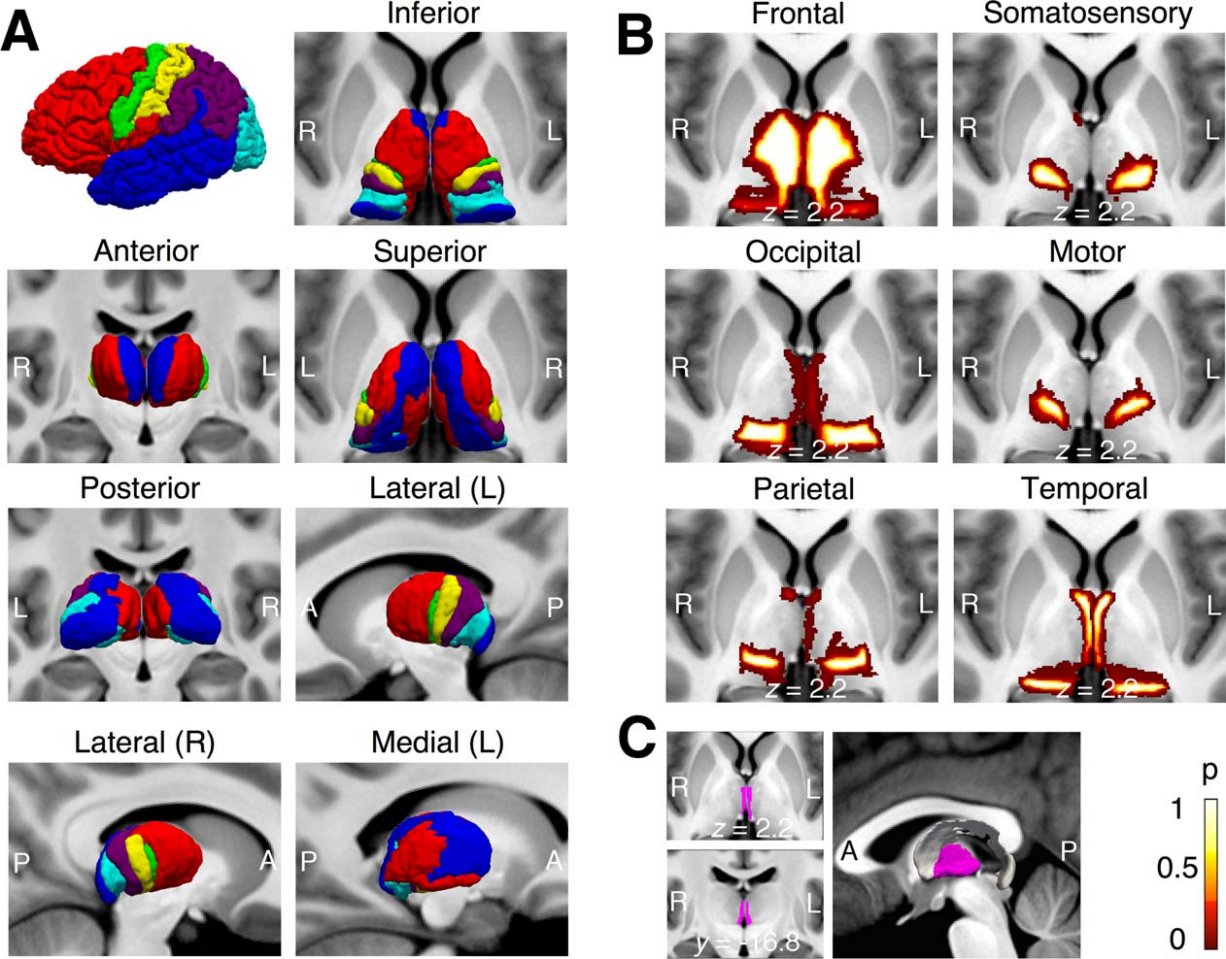
***A*:** Cortical masks for thalamus segmentation, based on FreeSurfer cortical parcellation (upper left panel, example subject). The 3D rendered maximum probability maps of corresponding group‐level thalamus segmentations in MNI space (thresholded at *P =* 0.3). ***B*:** Unthresholded probability maps of group‐level thalamus segmentations in MNI space. ***C*:** Axial and coronal views of group‐level paraventricular mask in MNI space on the left. 3D visualization of group‐level PVT mask (purple) and temporal thalamic segmentation (thresholded at *P =* 0.3).

Thalamus boundaries were extracted from the automated cortical and subcortical FreeSurfer segmentation. Connectivity‐based segmentation of the thalamus was based on voxel‐to‐region connectivity with frontal, parietal, occipital, temporal, motor and somatosensory cortex [Behrens et al., 2003a]. The six cortical masks were merged from respective labels in the FreeSurfer segmentation (see Fig. 2A). Tractography was then performed between the thalamus mask as seed, and the cortical masks as (classification) targets using probtrackx with default settings. A termination mask stopping pathways as soon as they entered the cortex, brainstem or cerebellum was employed to discard cortico‐cortical and other indirect tracts. A midline was also included in this mask, limiting tractography to the ipsilateral hemisphere. The thalamus was then parcellated using the FSL function find_the_biggest, which classifies each seed voxel according to the target area with which it has the highest probability of being connected (Fig. 2AB).

#### Definition of paraventricular thalamus

The thalamic region connecting mostly to temporal lobe was averaged across subjects in MNI space, thresholded at *P* = 0.15 (using FSL function *fslmaths*) and binarised. The resulting group‐level mask corresponded anatomically to dorsal parts of the medial and inferior pulvinar, superior parts of the mediodorsal nucleus, anterior complex and paraventricular thalamus (PVT). The PVT region was spatially contiguous with the other regions only in its anterior aspect. Thus voxels not belonging to PVT could be manually removed in comparison with schematic tables of an anatomical atlas by Mai et al. [2016] (Fig. 2C). To this end, we conservatively excluded possible non‐PVT voxels at the less well‐defined border to the anterior complex. Subsequently, this group‐level mask was transformed back into individual space.

### Statistical Analysis

Statistical analysis of connection strength was done in SPSS (version 22). Probabilistic tractography measures the number of traces arriving from each amygdala subregion at each target region [Behrens et al., 2003a]. We report this number as connection strength. Since connection strength depends on the size and thereby on the precise definition of the target region, we also report connection density as average number of traces arriving at each voxel of the target (connection strength/number of voxels in target mask). These values were analysed in a 2 (seed) × 6/74 (thalamic/cortical target) × 2 (hemisphere) ANOVA for connections to thalamus/cortex and a 2 (seed) × 2 (hemisphere) ANOVA for connections to PVT. Because connections to neighboring areas are not independent from each other, implying possible violations of multisphericity, Greenhouse–Geisser corrected *P*‐values are reported for within‐subject effects (Table 1). Post‐hoc analysis to localize significant ANOVA results was performed using paired t‐tests. Additionally, in Figures 5 and 6 we show voxel‐wise connectivity maps, which better depict the true connectivity gradients within cortical regions. Detailed region‐ and voxel‐wise statistics as well as probability maps for the amygdala clusters are available on https://doi.org/10.5281/zenodo.570535.

**Table 1:** Within‐subject ANOVA for connection strength and density between amygdala and thalamus or amygdala and cortex

	Connection strength		Connection density	
	*F*(df1,df2)	*P*	*F*(df1,df2)	*P*
Connectivity with thalamus
Seed	1.82 (1,49)	0.183	10.63 (1,49)	0.002 **
Target	73.87 (1.49,73.08)	<0.001 ***	135.39 (1.15,56.54)	<0.001 ***
Hemisphere	6.46 (1,49)	0.014 *	0.01 (1,49)	0.917
Seed × target	4.47 (2.15,105.15)	0.012 *	4.59 (1.23,60.19)	0.029 *
Seed × hemisphere	0.91 (1,49)	0.344	0.94 (1,49)	0.337
Target × hemisphere	1.38 (1.55,76.11)	0.254	1.70 (1.22,59.59)	0.198
Seed × target × hemisphere	2.26 (1.94,94.80)	0.111	0.85 (1.16,56.78)	0.377
Connectivity with cortex
Seed	230.53 (1,49)	<0.001 ***	119.38 (1,49)	<0.001 ***
Target	192.10 (1.44, 70.68)	<0.001 ***	157.85 (2.58,126.21)	<0.001 ***
Hemisphere	2.74 (1,49)	0.104	22.38 (1,49)	<0.001 ***
Seed × target	182.07 (1.82, 89.16)	<0.001 ***	130.08 (2.87,140.50)	<0.001 ***
Seed × hemisphere	0.59 (1,49)	0.445	1.31 (1,49)	0.258
Target × hemisphere	6.15 (1.70, 83.38)	0.005 **	7.37 (3.26,159.82)	<0.001 ***
Seed × target × hemisphere	4.73 (1.86, 91.02)	0.013 *	2.01 (3.10,151.54)	0.113

All *P*‐values are based on Greenhouse–Geisser corrected degrees of freedom to account for violations of multisphericity.

## RESULTS

### Amygdala Connections with Thalamus

**Figure 3:**
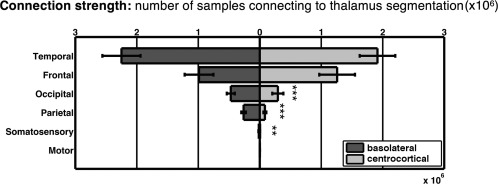
Mean [± SEM] connection strength from basolateral and centrocortical amygdala cluster to thalamic parcellations. ANOVA in Table I. Post‐hoc *t*‐tests: ** *P <* 0.01; *** *P <* 0.001.

**Figure 4:**
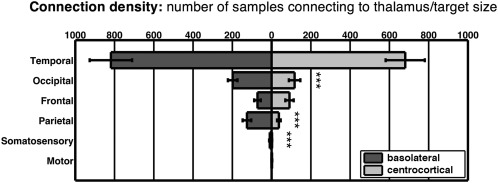
Mean [± SEM] connection density from basolateral and centrocortical amygdala cluster to thalamic parcellations (i.e., connection strength corrected for target size). ANOVA in Table I. Post‐hoc *t*‐tests * *P <* 0.05; ** *P <* 0.01; *** *P <* 0.001.

Thalamic regions differed in their connectivity to amygdala. Both amygdala nuclei had highest connectivity with the temporal parcellation (Figs. 3 and 4; tract maps in Supporting Information Figs. S1 and S2). Anatomically, this parcellation corresponds to dorsal parts of the medial and inferior pulvinar, superior parts of the mediodorsal nucleus, to the PVT and to parts of the anterior complex. Intermediate connection strength, in descending order, was found for frontal (MD/VA/VLa/anterior complex), occipital (parts of inferior pulvinar) and parietal (anterior pulvinar) thalamic parcellations (Fig. 3). Somatosensory (LP/VPL) and motor (VLp) segments showed low connection strength with amygdala. Relative connection density was markedly lower than connection strength specifically for the frontal thalamic parcellation (Fig. 4).

Connectivity profiles to the six thalamic segmentations were shown to be different for the two amygdala subregions in the ANOVA (seed × target interaction, see Table 1). Specifically, connection strength and density with the occipital and parietal thalamic parcellation, that is, inferior and anterior parts of the pulvinar, was significantly stronger for basolateral as compared with centrocortical amygdala (voxel‐by‐voxel connectivity analysis in Supporting Information Figs. S3 and S4).

Following automated thalamus parcellation, we delineated the PVT manually on the group level, and specifically assessed amygdala–PVT projections. Connection strength and density with PVT was stronger for the basolateral than centrocortical amygdala [strength: *F*(1,49) = 23.055, *P <* 0.001; density: *F*(1,49) = 21.183, *P <* 0.001]. Across both amygdala regions, connection strength and density with PVT was stronger in the left than right hemisphere [strength: *F*(1,49) = 20.365, *P <* 0.001; density: *F*(1,49) = 5.244, *P =* 0.026]. A significant seed × hemisphere interaction was seen in connection strength and density between amygdala and PVT [strength: *F*(1,49) = 8.052, *P =* 0.007; density: *F*(1,49) = 4.669, *P =* 0.036].

### Amygdala Connections with Cortex

**Figure 5:**
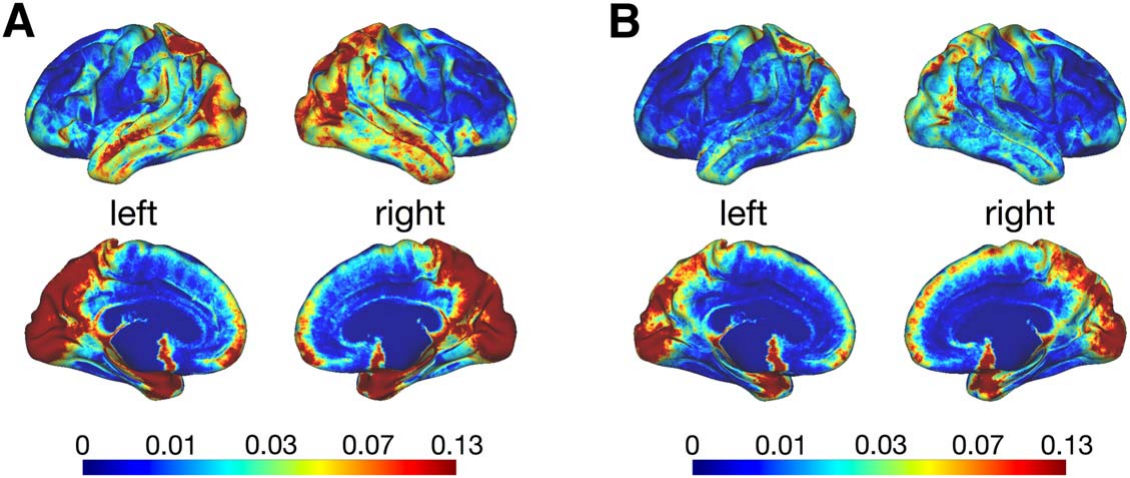
Voxel‐by‐voxel analysis of mean connection strength between ***A*:** basolateral or ***B*:** centrocortical amygdala cluster and cortical surface. Color‐coding is on logarithmic scale.

**Figure 6:**
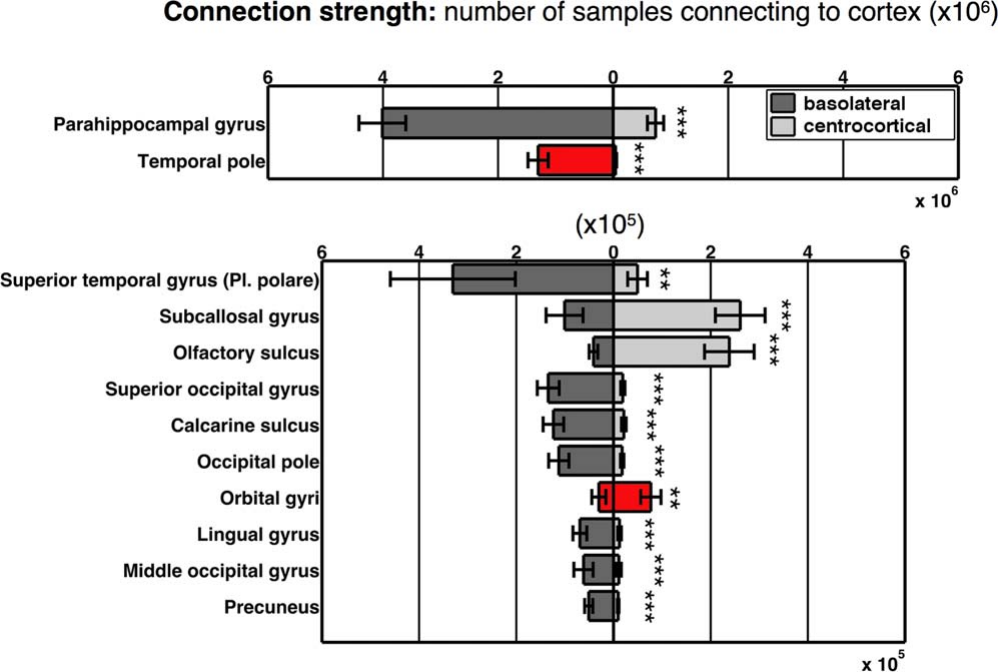
Mean [± SEM] connection strength from basolateral and centrocortical amygdala cluster to cortex. The 12 cortical areas with the highest connection strength to the amygdala are shown. Cortex areas used in amygdala parcellation (i.e., OFC/TP) are marked red. ANOVA in Table I. Post‐hoc *t*‐tests, * *P <* 0.05; ** *P <* 0.01; *** *P <* 0.001. [Color figure can be viewed at http://wileyonlinelibrary.com]

**Figure 7:**
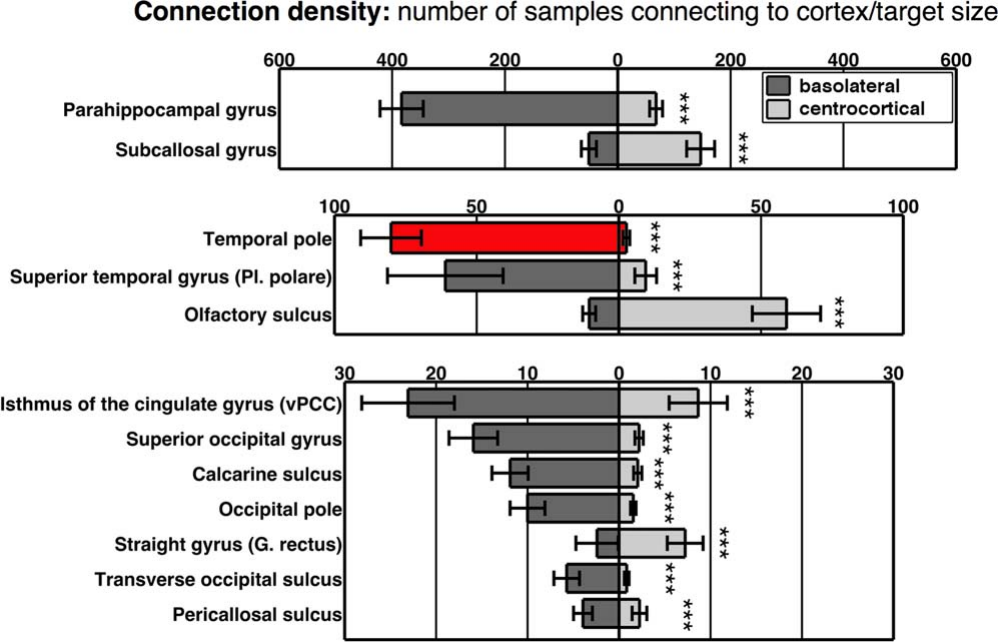
Mean [± SEM] connection density from basolateral and centrocortical amygdala cluster to cortex (i.e. connection strength corrected for target size). The 12 cortical areas with the highest connectivity to the amygdala are shown. Cortex areas used in amygdala parcellation (i.e., OFC/TP) are marked red. ANOVA in Table 1. Post‐hoc *t*‐tests, * *P <* 0.05; ** *P <* 0.01; *** *P <* 0.001. [Color figure can be viewed at http://wileyonlinelibrary.com]

Deep and superficial amygdala clusters displayed distinct and widespread connections to the cortex (Figs. 5, 6, 7). As seen in Table 1, connectivity profiles of the two clusters were different both in connection strength and density (seed × target interaction). We observed greater connection strength between basolateral amygdala and temporal/occipital cortical regions such as superior temporal cortex or calcarine sulcus, as well as greater connection strength between centrocortical amygdala and prefrontal regions such as subcallosal gyrus, olfactory sulcus and straight gyrus (Figs. 5 and 6). Results for connection density are shown in Figure 7. We also found a significant target × hemisphere interaction in connection strength and density. Parahippocampal gyrus, temporal pole, superior temporal gyrus, subcallosal gyrus, olfactory sulcus, superior occipital gyrus and calcarine sulcus, in descending order, showed highest connection strength with the entire amygdala (i.e., summing across amygdala nuclei). In addition, we found a less pronounced but conceivably important connection with the anterior insular cortex.

## DISCUSSION

We investigated in vivo subregional connectivity profiles for amygdala nucleus groups with thalamic and cortical regions. The following key findings emerged. First, we were able to provide, to the best of our knowledge, the first description of a direct connection between amygdala and paraventricular thalamus (PVT) in humans, which was stronger for the basolateral amygdala. Moreover, we demonstrate substantial connectivity of the amygdala with the pulvinar, relative to other thalamic regions. This connection was more pronounced for basolateral than centrocortical amygdala. Second, we show that amygdala subregions have widespread but distinct connectivity profiles to the cortex. While the basolateral amygdala seems to be predominantly connected with temporal and occipital regions and have more connections overall, the centrocortical amygdala displays more connectivity with the frontal lobe.

Connections between PVT and amygdala have so far not been investigated in humans. In rodents, the PVT projects mainly to central amygdala and has weaker connections to basolateral nuclei [Li and Kirouac, 2008; Penzo et al., 2015; Vertes and Hoover, 2008] while in macaque monkeys it displayed strong reciprocal connections with basal amygdala and weaker connections with central amygdala [Hsu and Price, 2009]. However the projections to central amygdala in rats arise mainly from posterior parts of the PVT, which were not injected in the study by Hsu and Price [2009]. The authors further report that an anterograde tracer injection in the central amygdala did indeed result in labelling of a strong projection to posterior PVT. Nevertheless, our data suggest significantly stronger connections with basolateral than centrocortical amygdala in humans. The PVT is activated by exposure to food rewards, addictive drugs and various stressors in experimental animals and has been implicated in positive and negative emotional responses [Kirouac, 2015]. A recent optogenetic study has shown the PVT to mediate opiate dependency in interaction with nucleus accumbens [Zhu et al., 2016], while excitation of nucleus accumbens by basolateral amygdala facilitates reward‐seeking behaviour in mice [Stuber et al., 2011]. It seems plausible to consider direct interaction between basolateral amygdala and PVT as part of a regulatory circuit in this context that deserves further investigation in the context of neuropsychiatric conditions.

Projections from pulvinar to the amygdala have been reported in non‐human primates [Aggleton et al., 1980; Day‐Brown et al., 2010; Jones and Burton, 1976; Romanski et al., 1997] and humans [Tamietto et al., 2012]. Mehler [1980] however explicitly did not reveal such connections in rhesus and squirrel monkeys. Some have argued that evidence from non‐human primate studies supports the existence of connections only between amygdala and medial pulvinar, which is multimodal in function and not primarily ‘visual’, but not between amygdala and inferior pulvinar, which is strongly interconnected with the visual cortex [Pessoa and Adolphs, 2010]. This view has been disputed [De Gelder et al., 2011] based on more recent anatomical evidence for amygdala connection with the visual pulvinar in tree shrews [Day‐Brown et al., 2010] and humans [Tamietto and De Gelder, 2010], later replicated in Rafal et al. [2015]. Our data support this latter view and suggest connections of the amygdala both with the temporal parcellation of the thalamus, including dorsal parts of the medial and inferior pulvinar, and with the occipital parcellation, which corresponded anatomically to parts of the inferior pulvinar. Since this thalamic parcellation is defined by connections with the visual cortex, our data clearly suggest amygdala connections with visual pulvinar. Thus, we provide further evidence for the existence of a direct pathway between the amygdala and visual pulvinar in humans. Crucially, this connection is more pronounced for the basolateral than the centrocortical amygdala. The existence of a direct subcortical visual route between amygdala and visual pulvinar would enable attribution of emotional salience to coarse visual information, before it is integrated in the neocortex and reaches conscious awareness [Vuilleumier, 2005]. In this context, a direct pathway in primates between amygdala and the thalamic reticular nucleus (which gates thalamo‐cortical signalling), has also been suggested to mediate selective attention based on emotional information [John et al., 2016; Zikopoulos and Barbas, 2012] and would merit further investigation in humans.

Consistent with observations in the cynomolgus monkey [Aggleton and Mishkin, 1984] our data furthermore suggest substantial connectivity between amygdala and mediodorsal thalamus, which is mainly included in the frontal and to a small extent in the temporal thalamic segmentation.

Strikingly, based mainly on rodent and cat studies, McDonald [1998] reported a very specific architecture of amygdala–cortical connections. In particular, the amygdala appears to receive projections from all sensory modalities, but only primary cortex of olfactory, gustatory and visceral modality connect to the amygdala directly; in the auditory, visual and somatosensory domain, tertiary and polymodal cortices project to the amygdala in modality‐specific cascades. Early auditory information is furthermore directly conveyed to the thalamus via amygdala [Romanski and LeDoux, 1992], while, as discussed above, direct connections between amygdala and pulvinar have also been suggested as part of a potential alerting system [LeDoux, 2000]. In line with this view, our data suggest amygdala connections with the primary gustatory cortex, which is partially situated in the anterior insula. Furthermore we found strong connectivity, predominantly of centrocortical amygdala, with olfactory sulcus, which may target the herein encapsulated olfactory tract. Also consistent with the proposed model, we did not find any substantial amygdala connections with primary somatosensory cortex, but connections with precuneus and to a lesser extent the superior parietal lobule, which contain the somatosensory association cortex. As suggested by McDonald [1998] we did not find connections to core auditory cortex. In the last two decades a dual stream organization of the auditory cortex has been proposed first in non‐human primates and was then adopted in humans [DeWitt and Rauschecker, 2013]. Here we remarkably only found connections with the anterior part of the superior temporal gyrus (planum polare), which is part of the ventral auditory stream and has been recently implicated in auditory single‐word comprehension [DeWitt and Rauschecker, 2015]. The connections were significantly stronger for the basolateral amygdala. Connections to the posterior auditory stream, that is, planum temporale of the superior temporal gyrus were not found. This is also consistent with a recent observation that emotional augmentation of neural activity during auditory stimulus localization, presumably through interaction with the amygdala, exists in the ventral but not posterior auditory stream [Kryklywy et al., 2013].

According to McDonald [1998] projections of the occipital lobe to the amygdala do not exist in the monkey. In contrast direct efferent connections from the amygdala to V1 have been described in non‐human primates [Amaral and Price, 1984; Mizuno et al., 1981; Tigges et al., 1982; Tigges et al., 1983]. Here we did find strong connections of basolateral amygdala with primary visual cortex V1, which is situated in the occipital pole and pericalcarine sulcus, as well as with higher order visual processing areas, such as lingual gyrus and transverse occipital sulcus. Since there is no information on directionality in tractography, it is not possible to infer if these connections are efferent or afferent. Assuming however that this projection is indeed arising from the amygdala, as evidence from primates suggests, connections between basolateral amygdala and V1 in combination with the aforementioned interaction between amygdala and visual pulvinar would further consolidate a two‐pathway hypothesis for emotional control of visual perception, which propagates early processing in the pulvinar–amygdala pathway and a feedback projection from amygdala to visual cortex [Vuilleumier, 2005].

Both amygdala subregions connected most strongly with the parahippocampal gyrus, which includes the polymodal entorhinal and perirhinal cortex. These structures together with the hippocampus form the medial temporal lobe system, which is essential to memory processing [Eichenbaum et al., 2007; Squire and Zola‐Morgan, 1991] and spatial orientation [Epstein et al., 1999; Fyhn et al., 2004; O'Keefe and Dostrovsky, 1971]. Amygdala connections to other polymodal areas such as the temporal pole and prefrontal cortices were also found to be robust and subregionally distinct. However, we note that our amygdala parcellation was based on connections to temporal pole and orbitofrontal cortex in the first place, such that connections to these two regions should be regarded with caution and have limited interpretability.

Additionally we were able to show connections of the basolateral amygdala with the isthmus of the cingulate gyrus and the adjoining precuneus, which has been proposed as a functional core of the default‐mode network [Utevsky et al., 2014] as well as connections of centrocortical amygdala with primary motor cortex in accord with findings of Grezes et al. [2014].

Alterations of structural connectivity between limbic and prefrontal brain regions have been associated with general anxiety disorder [Cha et al., 2016; Tromp et al., 2012] while structural connectivity of amygdala with prefrontal cortex as well as more complex cortical networks can predict trait anxiety in humans [Greening and Mitchell, 2015; Kim and Whalen, 2009]. Interestingly deficits of white‐matter integrity in amygdala–prefrontal pathways have also been found in Williams syndrome [Avery et al., 2012], a neurodevelopmental disorder associated with significantly high non‐social fears. Autistic traits in healthy humans have been associated with increases of white‐matter connectivity between amygdala and superior temporal sulcus [Iidaka et al., 2012], an area involved in face processing. Increased left amygdala connectivity with hippocampus, cerebellum and brainstem has been shown for patients in remission from major depressive disorder (MDD) [Arnold et al., 2012], while a specific decrease in white‐matter integrity of the right solitary tract, which connects brainstem to amygdala, has been reported in MDD‐patients [Song et al., 2014]. Abnormalities in structural connectivity have been furthermore found in bipolar disease [Houenou et al., 2007; Wang et al., 2009], conduct disorder [Passamonti et al., 2012] and after prenatal cocaine exposure [Li et al., 2013].

Most of these existing studies of structural amygdala connectivity however have either used coarse automated masks of the amygdala; masks based on functional amygdala activation, or have analysed the integrity of the uncinate fascicle as a proxy of amygdalo‐frontal connectivity. More refined studies of subregional amygdala connectivity in the context of neuropsychiatric disease appear to be missing. Our comprehensive connectivity profiles of the human amygdala may guide future work related to normal and pathological function, which could build on the same methods.

Limitations of our study include inherent difficulties of tractography such as the unknown directionality of connections. There is an ill‐defined influence of distance on tractography results. Tractography has furthermore been known to yield false positive results concerning anatomical connections due to diverse physical and biological factors [Thomas et al., 2014]. We restricted our analysis to cortical and thalamic connections of the amygdala; the spatial resolution of our data set did not allow delineating connections to small subcortical structures [Kamali et al., 2015]. Moreover, since we parcellated the amygdala based on voxel‐to‐voxel connectivity with orbitofrontal cortex and temporal pole, connectivity profiles for individual amygdala clusters with these regions are confounded and must be interpreted with caution. However this does not affect connections to other cortical and thalamic regions. It would be of great interest to investigate even finer amygdala subdivisions and their respective connections. In particular, separating the central from the cortical nucleus would merit further exploration. The intercalated nuclear complex of the amygdala, a group of inhibitory neurons, which form a net in between major amygdala nuclei, intrinsically regulate amygdala activity [Zikopoulos et al., 2016] and are critically involved in fear extinction [Likhtik et al., 2008] represent another important target for future research that may benefit from a more advanced amygdala parcellation method. However, while a recent study has proposed a probabilistic atlas of major nuclei based on high‐resolution group data [Tyszka and Pauli, 2016], a robust method for amygdala parcellation into smaller substructures on an individual level is still missing. Finally, since we focused on white‐matter connectivity, our approach was blind to direct amygdala–hippocampal connections, important for contextual conditioning [Marschner et al., 2008] or anxiety‐like behaviour [Bach et al., 2014; Korn et al., 2017]. A further limitation of our study is that the thalamus boundaries obtained from FreeSurfer did not include lateral and medial geniculate nuclei. Finally, as has been demonstrated for the sub‐thalamic nucleus using tracing techniques in non‐human primates [Haynes and Haber, 2013], brain connectivity is not structured across sharp anatomical borders but rather by connectivity gradients. The amygdala parcellations that we rely in our probabilistic tractography approach do not fully reflect this factor. A recently developed segmentation method of the thalamus based on probabilistic tractography characterized by Euclidian distance and connectivity gradients [Lambert et al., 2016] may constitute a worthwhile extension to our work and partially address this limitation in case of the thalamus.

## CONCLUSION

In conclusion, we were able to provide evidence of substantial and direct connectivity between amygdala and subdivisions of the pulvinar. Here connections with inferior and anterior parts of the pulvinar were shown to be stronger for the basolateral amygdala.

Furthermore we were able to delineate the PVT and found evidence of connection with both amygdala subregions, predominantly the basolateral subregion. Our results consolidate and expand existing evidence of direct anatomical interaction between amygdala and thalamus in humans.

Finally we established detailed connectivity profiles for basolateral and centrocortical amygdala nuclei to the cortex in humans. Connections to cortex proved to be extensive and distinct for both subregions, and were largely consistent with findings from studies in non‐human animals.

We hope that our results may be used as a guide for functional and pathophysiological neuroimaging studies.

## FINANCIAL DISCLOSURES

The authors declare no competing financial interests.

## Supporting information

Supporting Information
