# Supplementary material for: Deconstructing white matter connectivity of human amygdala nuclei with thalamus and cortex subdivisions in vivo
Source: Hum Brain Mapp. 2017 May 17;38(8):3927–40. doi: 10.1002/hbm.23639 (PMC5729634; doi:10.1002/hbm.23639)
Supplement: Supplementary file 1 — Supporting Information [file HBM-38-3927-s001.pdf]

Supporting information

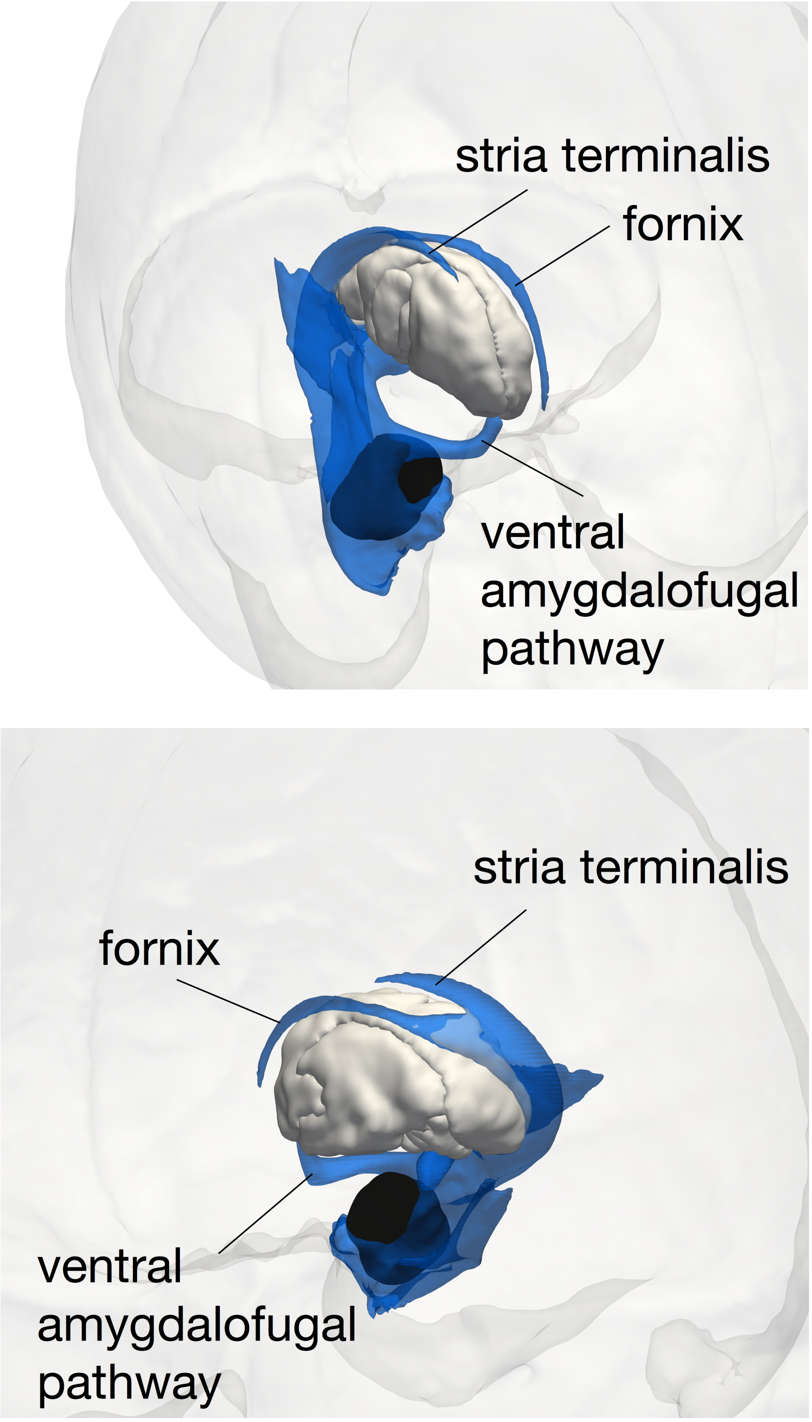

Figure S1: Thresholded tract maps. Maps are averaged across participants (n = 50), thresholded at 0.5‰ of total number of samples, 3D-rendered to show tracts between basolateral amygdala and thalamus parcellations.

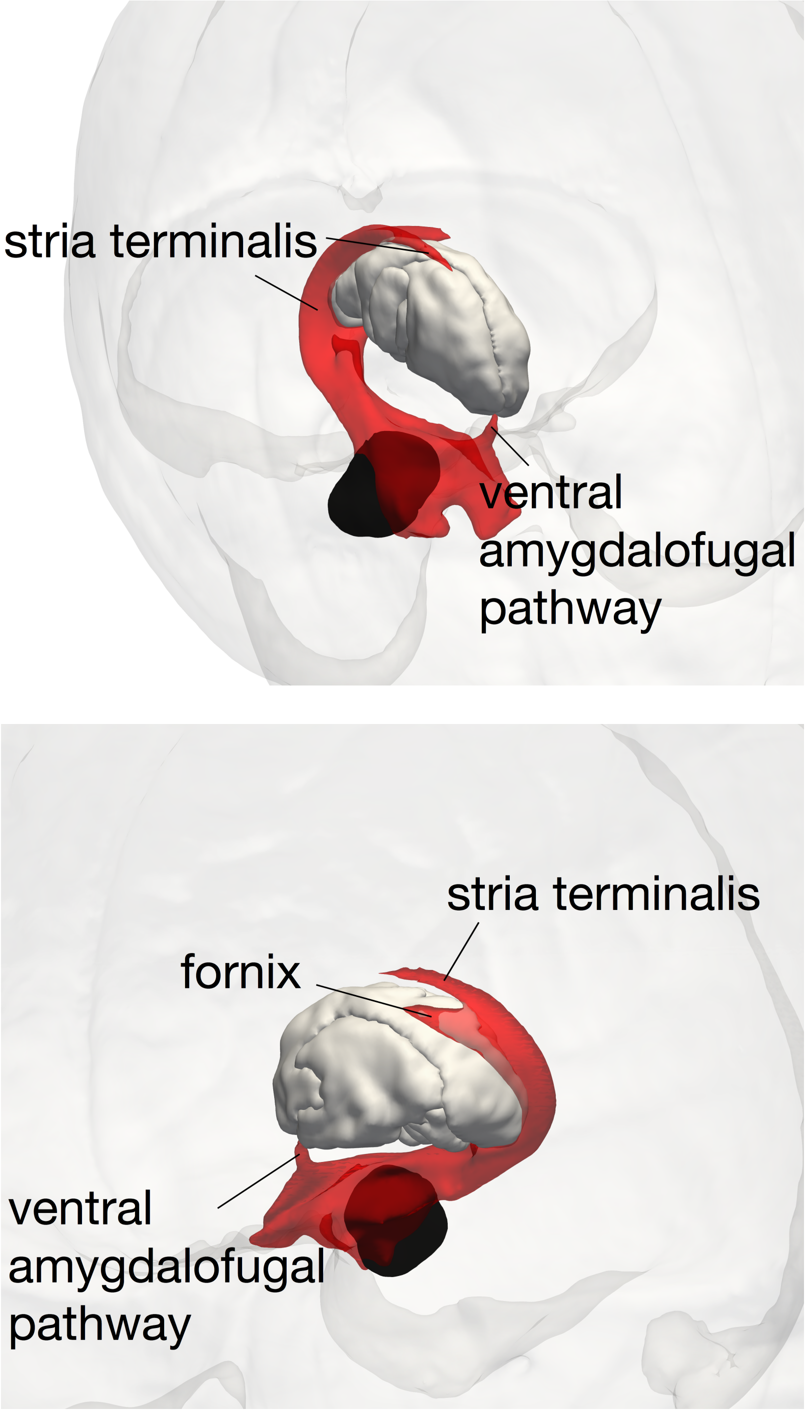

Figure S2: Thresholded tract maps. Maps are averaged across participants (n = 50), thresholded at 0.5‰ of total number of samples, 3D-rendered to show tracts between centrocortical amygdala and thalamus parcellations.

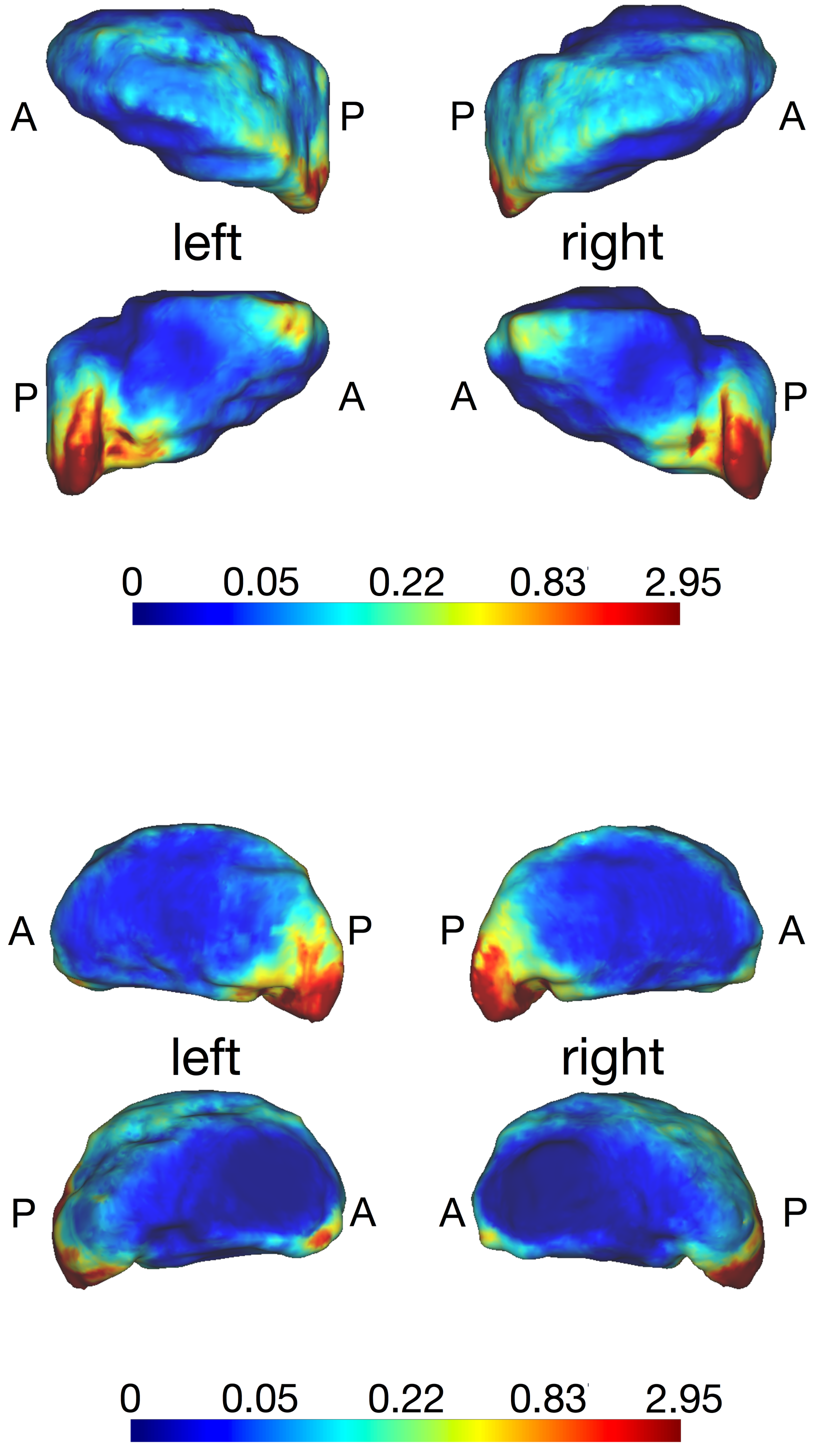

Figure S3: Voxel-by-voxel analysis of mean connection strength between basolateral amygdala cluster and thalamus surface (n=50). Superior and inferior views are seen in the upper panel; lateral and medial views in the lower panel. Logarithmic scale was used.

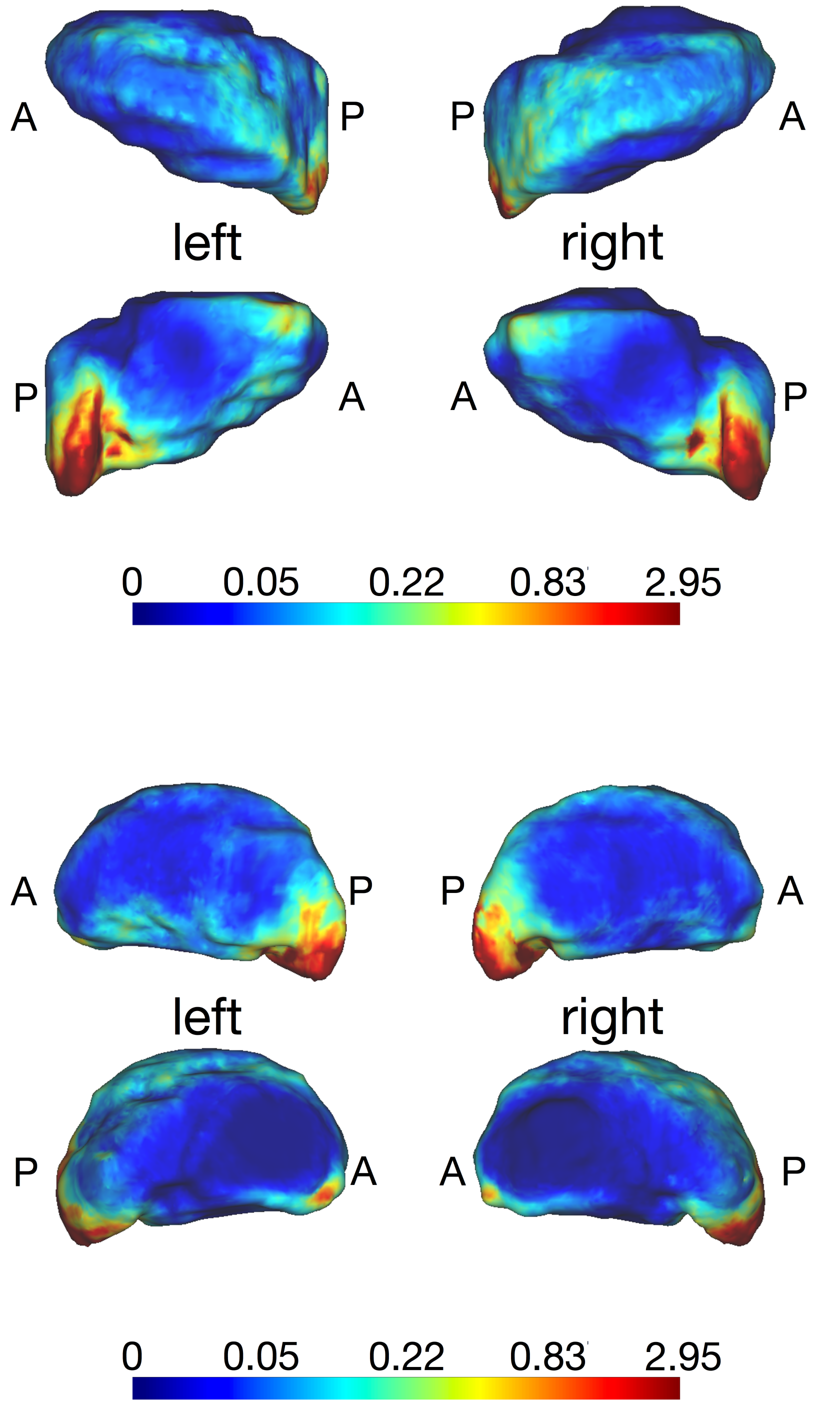

Figure S4: Voxel-by-voxel analysis of mean connection strength between centrocortical amygdala cluster and thalamus surface (n=50). Superior and inferior views are seen in the upper panel; lateral and medial views in the lower panel. Logarithmic scale was used.
